# Non-codified traditional medicine practices from Belgaum Region in Southern India: present scenario

**DOI:** 10.1186/1746-4269-10-49

**Published:** 2014-06-16

**Authors:** Vinayak Upadhya, Harsha V Hegde, Shripad Bhat, Sanjiva D Kholkute

**Affiliations:** 1 Regional Medical Research Centre, Indian Council of Medical Research, Nehru Nagar, Belgaum 590010, India

**Keywords:** Belgaum, Convenience sampling, Disease diagnosis, Ethnomedicine, Non-codified medicine, Preference ranking, Sharing of knowledge, Traditional medicine, Traditional practitioner, Western Ghats

## Abstract

**Background:**

Traditional medicine in India can be classified into codified (Ayurveda, Unani, Siddha, Homeopathy) and non-codified (folk medicine) systems. Both the systems contributing equally to the primary healthcare in India. The present study is aimed to understand the current scenario of medicinal practices of non-codified system of traditional medicine in Belgaum region, India.

**Methods:**

The study has been conducted as a basic survey of identified non-codified traditional practitioners by convenience sampling with semi structured, open ended interviews and discussions. The learning process, disease diagnosis, treatment, remuneration, sharing of knowledge and socio-demographic data was collected, analysed and discussed.

**Results:**

One hundred and forty traditional practitioners were identified and interviewed for the present study. These practitioners are locally known as “Vaidya”. The study revealed that the non-codified healthcare tradition is practiced mainly by elderly persons in the age group of 61 years and above (40%). 73% of the practitioners learnt the tradition from their forefathers, and 19% of practitioners developed their own practices through experimentation, reading and learning. 20% of the practitioners follow distinctive “Nadi Pariksha” (pulse examination) for disease diagnosis, while others follow bodily symptoms and complaints. 29% of the traditional practitioners do not charge anything, while 59% practitioners receive money as remuneration.

Plant and animal materials are used as sources of medicines, with a variety of preparation methods. The preference ranking test revealed higher education and migration from villages are the main reasons for decreasing interest amongst the younger generation, while deforestation emerged as the main cause of medicinal plants depletion.

**Conclusion:**

Patrilineal transfer of the knowledge to younger generation was observed in Belgaum region. The observed resemblance in disease diagnosis, plant collection and processing between non-codified traditional system of medicine and Ayurveda require further methodical studies to establish the relationship between the two on a more objective basis. However, the practice appears to be at crossroads with threat of extinction, because of non-inheritance of the knowledge and non-availability of medicinal plants. Hence conservation strategies for both knowledge and resources at societal, scientific and legislative levels are urgently required to preserve the traditional wisdom.

## Background

Traditional medicine, as defined by WHO, include diverse health practices, approaches, knowledge and beliefs incorporating plant, animal, and/or mineral based medicines, spiritual therapies, manual techniques and exercises, applied singly or in combination to maintain well-being, as well as to treat, diagnose or prevent illness [1]. Based on this statement, traditional medicine and practices can be classified broadly into (a) traditional medicine with a systematic codified body of knowledge either in the form of pharmacopoeias, or ancient scriptures like Ayurveda, Chinese and Tibetan medicine, Siddha, Unani etc.; (b) non-codified system of traditional medicine or folk medicine, which is transmitted by oral means and is mostly acquired through trial-and-error approaches; (c) spiritual or shamanistic medicine, which has a strong religious/spiritual element and is practiced only by highly specialized local experts [2,3] and (d) allied forms of health knowledge such as Yoga, Tai-Chi, various forms of meditation and breathing techniques, massage techniques etc. [4].

All the above types of traditional medicine practices are found in India, which takes care of the primary health needs of about 70% of the Indian population [1]. Among them, the non-codified and codified systems of traditional medicine share the equal task in managing primary health care [4,5]. The non-codified system of traditional medicine is diverse and varies with geography, local flora and culture. It was developed in accordance with primary needs and locally available resources of a particular region. The system differs from one region to the other and is known by various names like indigenous medicine, ethno medicine, bush medicine, little traditions, folk/folklore medicine and many more. However, the practice is not formalized in many countries and to a large extent remains in non-codified form [4]. This system is diminishing with time due to various factors like inadequate support from the legislation [3,4]. Therefore, proper documentation is needed to preserve this ancient, non-codified traditional knowledge.

In India, attempts have been made to document the ethnobotanical wealth since 17^th^ century, initiated by a Dutch governor Hendrik Adriaan Van Rheede tot Draakenstein [6] and others [7,8]. However, organized ethnobotanical studies were intensified much later, with contributions of researchers like Janaki Ammal and SK Jain [9]. The works of these authors encouraged scientists from India to take up scientific studies and now documentation is available from every state in India [9-11]. Research publications are also available from Belgaum [12,13] and from other parts of Karnataka state [14-26], which mainly concentrate on documentation of medicinal plants [23], formulations and usages (disease) from specific tribes [25,26] and/or practices in restricted geographical areas [12-22,24]. The status of traditional practice provide the basis for further management and conservation strategies of the system, hence the present study was undertaken to document these practices in general and to understand the current scenario from Belgaum region of India. Further, this article is also aimed to initiate the thought process towards recognizing, safeguarding and propagating the age old treasure of non-codified traditional medicine practices.

## Methods

### Study area

**Figure 1:**
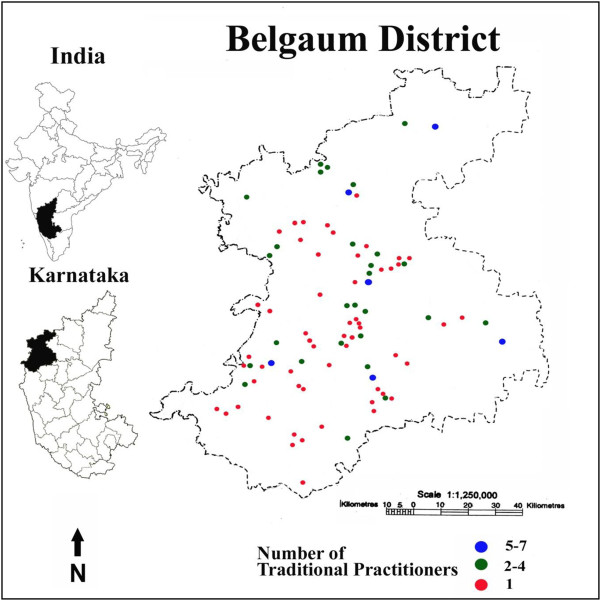
Map showing traditional practitioners from Belgaum District.

Belgaum district is situated in the North-West part of Karnataka state in India (15°23 to 16°58’ N. and 74°5’ to 75°28’ E) spreads over an area of 13, 415 sq.kms sharing state borders with the states of Maharashtra and Goa (Figure 1). Topographically the district can be divided into hilly area, semi-hilly area and plains with varied rainfall gradient and vegetation types along the line of the Western Ghats - a biodiversity hot spot [27]. Belgaum district has a unique cultural amalgamation of adjacent states blended with native Kannada culture. It has a total population of 4.778 million as per 2011 census. About 75% of the total population resides in rural areas and mainly depend on agriculture for their livelihood [28]. People belonging to various religions, castes and tribes reside in the area enriching the ethnic diversity.

### Field survey

#### ***Identification of traditional practitioners***

The data collection method involved eliciting preliminary data from the traditional practitioners. A list of traditional practitioners in the study area was prepared by convenience sampling, through discussions with various social stakeholders like local Non-Government Organizations, staff of primary health centres, village chiefs, raw drug vendors, school teachers, villagers and patients visiting the traditional practitioners. Efforts were made to represent all parts of the district. The definition provided by WHO (1976) was used as basic criteria to identify the traditional practitioner [29]. The issues verified for considering practitioners as informants in this study were (a) recognition by the local public in his/her area and utilization of his/her services (b) treatments based on plants and/or animal substances, or methods based on religious and cultural aspects and (c) willing to discuss the general practices and status of traditional medicine.

However, practitioners were not compelled to reveal the exact formulations and secrets of their practice. Help of local persons was taken to develop rapport with the traditional practitioners for the first interaction. Practitioners belonging to communities like Agasa, Badiger, Banjara, Bhois, Hanabaru, Holaya, Kurubar, Kammar, Lad, Lingayata and Maratha participated in the present study. However, collection of information on race and caste was avoided because of associated social stigma. Nonetheless, care was taken that the objective of the study was not affected. The non-codified traditional practitioners have been referred to as traditional practitioners and the term traditional medicine refers to non-codified traditional medicine, throughout this article.

### Semi structured, open-ended interviews

Ethical approval for this research was obtained from Ethics Committee on Human Subjects Research, KLE University, Belgaum (KLEU/ D 6632-6635). The aim and objectives of the study was explained to the traditional practitioners in the local language by the authors and consent was obtained. The traditional practitioners were visited repeatedly (minimum four to five visits, with a gap of one to two months between each visit) to collect the information. Each visit lasted between two to four hours at the practitioner’s home or work place. Both qualitative and quantitative data were gathered using semi-structured questionnaire (provided as Additional file 1) and open ended interview as the principle tool as per the method of Martin [30] and Jain [10,11]. The interactions were initiated by questioning about the socio-demographic information of traditional practitioners, such as name, sex, age, literacy, religion and status of traditional medicine. Once the practitioner became familiar, queries on the practice were made. The semi-structured questionnaire was considered as a basic tool for interview. Questions were put to the practitioner from the questionnaire during the interview by the author. The interview was extended further as open ended discussion to document the details of the practice. The group discussion was conducted at practitioner’s place during the subsequent visits. The group involved local persons, village chiefs and patients present at that time along with traditional practitioner. The discussions were of informal nature. History of traditional practice, learning methods, remuneration, success and constraints for the practice were discussed. Qualitative data was collected through group discussions about the traditional practices [31].

### Preference ranking tests

Existence of traditional practice depends mainly on inheritance of the practice to successive generation and availability of medicinal plant resources. Preference ranking method was adopted for quantitative assessment of local significance about factors hindering practice [31]. Practitioners were asked to rank the reasons or causes according to their impact in relation to two different issues: 1) Non-availability of medicinal plants, and 2) Disinterest of younger generation in traditional practice. Five reasons were indicated for ranking by the authors (provided in Additional file 1). The ranks were assigned values, with most important as five, and least important being one. Scores were added and overall ranking was assigned for the reason in decreasing order of the total scores. The top ranked reason was considered as the major reason for diminishing practice.

### Data processing and analysis

Classification of age was made with intervals of ten years starting from 21 years to 71 years. Age above 61 years was considered as elderly age group. According to the census of India, Hinduism is the main religion followed by Islam. Remaining religions like Christianity, Jainism and Buddhism were included in other category. MS Excel 2007 and SPSS 13 were used for tabulation analysis. The results were presented as percentages, frequencies, cross-tabulation and graphs. Percent values are calculated as $\left[\frac{\mathrm{Nc}}{\mathrm{Nt}}\right]\times 100$. The terms used in the formula are defined as follows: Nc = Number of traditional practitioners in any category; Nt = Total number of traditional practitioners who participated in the study (Nt = 140). Calculated percent values were presented as the decimal numerals in tables and figures and as integers in the manuscript.

## Results and discussion

In all 140 traditional practitioners were identified and interviewed from all parts of the district (Figure 1). Information on non-codified system of traditional medicine, traditional practice and its current status were gathered. These traditional practitioners are known as “Vaidya” (means doctor) in Belgaum district as in other places of India [32-34].

### Socio-demographic characters

Socio-demographic data include age, gender, literacy rate and religion of traditional practitioners. The results are tabulated in Table 1.

**Table 1:** Number of traditional practitioners with demographic data

**Age**		**Gender**		**Literary rate**				**Religion**		
	**Nc**	**Male**	**Female**	**Illiterate**	**Primary**	**High school**	**Higher education**	**Hindu**	**Muslim**	**Christian, Jain and others**
	**(%)**	**Nc (%)**	**Nc (%)**	**Nc (%)**	**Nc (%)**	**Nc (%)**	**Nc (%)**	**Nc (%)**	**Nc (%)**	**Nc (%)**
**20-30**	5 (3.6%)	5 (3.6%)	Nil	Nil	Nil	3 (2.1%)	2 (1.4%)	4 (2.9%)	1 (0.7%)	Nil
**31-40**	18 (12.9%)	15 (10.7%)	3 (2.1%)	Nil	6 (4.3%)	9 (6.4%)	3 (2.1%)	13 (9.3%)	4 (2.9%)	1 (0.7%)
**41-50**	23 (16.4%)	20 (14.3%)	3 (2.1%)	2 (1.4%)	6 (4.3%)	9 (6.4%)	6 (4.3%)	18 (12.9%)	4 (2.9%)	1 (0.7%)
**51-60**	38 (27.1%)	34 (24.3%)	4 (2.9%)	3 (2.1%)	18 (12.9%)	4 (2.9%)	13 (9.3%)	30 (21.4%)	4 (2.9%)	4 (2.9%)
**61-70**	34 (24.3%)	31* (22.1%)	3 (2.1%)	18 (12.9%)	7 (5.0%)	5 (3.6%)	4 (2.9%)	30 (21.4%)	03 (2.1%)	1 (0.7%)
**71 and above**	22 (15.7%)	21* (15.00%)	1 (0.7%)	9 (6.4%)	8 (5.7%)	4 (2.9%)	1 (0.7%)	22 (15.7%)	Nil	Nil
**Total**	**140 (100.0%)**	**126 (90.0%)**	**14 (10.0%)**	**32 (22.9%)**	**45 (32.1%)**	**34 (24.3%)**	**29 (20.7%)**	**117 (83.6%)**	**16 (11.4%)**	**07 (5.00%)**

Nc – Number of traditional practitioners; (%) - Represent calculated percent value of traditional practitioner in each category; *Death of practitioner in the age group (3 each).

The study results showed that a large proportion of traditional practitioners were in the age group of 51–60 years (27%) followed by that of 61–70 years (24%). A significant number of traditional practitioners were found to be above 71 years of age (16%). This trend appears similar to that noticed in other studies and indicates decline in the number of practitioners as one goes down the age groups [35-38]. Discussion with the practitioners revealed the perception of lack of interest towards traditional practices in younger generation. During the study period, unfortunately, six practitioners died due to old age; in those cases discontinuation of tradition was observed in three because of non-inheritance of practice. This situation is obviously a threat to the continued survival of the traditional medicine as a good number of practitioners are old and may not be able to continue the practice for much longer, and in many cases do not have trained heirs.

The percentage of male and female traditional practitioners in the study area is 90% and 10% respectively. Earlier studies from the same region [12,14], within the country [35,36,38], and elsewhere have also suggested lower percentage of female practitioners [37,39,40]. The reason behind this could be the patriarchal society and patrilineal inheritance in this region, where medicinal practice is mainly passed on to male children who are considered heirs of the families.

Studies showed that 23% of traditional practitioners were illiterate and fall into the older age group of 61 years and above. The study results were in tune with the literacy rate of the district (74%) which is up to 77% [28]. However, only 21% of traditional practitioners had higher education up to bachelor’s level. It is pertinent to note that a large number of traditional practitioners had only primary education and left school once they learnt reading and writing. Several earlier studies also reported a low literacy rate among traditional practitioners [40,41]. This may be one of the reasons for non-documentation of traditional practices.

Results also indicated that 84% of the traditional practitioners belong to the Hindu religion, under various castes and tribes.

### General practice and status of traditional medicine

In India, non-codified traditional medicine had its impact on community healthcare for millennia. It has its own advantages as it shares the same culture and environment of the patients. Further, locally available resources are used and it is cost effective. The practice also has its own individuality from one place to another, and between practitioners, because of its inherent nature. Therefore, proper documentation is essential not only for preservation of the knowledge, but also for documenting the dynamic trend in practice.

### Learning

Present study reveals that traditional practitioners learnt the practice (Table 2) from their forefathers (73%), from others or through self experimentation (19%) or by reading books (9%). Similar type of learning trend by traditional practitioners was observed elsewhere in India [3,38], and other parts of the world [37,39,42]. In this study it was observed that the learning process is only through verbal communication without any documentation or written texts in all the cases. Skills and knowledge were acquired by the disciple while assisting their parents or grandparents in treating patients. On several occasions, the disciples started their own practice only after the death of the master. Practicing knowledge is believed to be sacred by several practitioners and the practicing secrets were disclosed only to a trusted younger member of the family.

**Table 2:** Learning of practice and disease diagnosis by traditional practitioners

**Learning of practice from**	**Number of practitioners (%)**
Forefathers	102 (72.9%)
Others or self experimentation	26 (18.6%)
Reading	12 (8.6%)
**Disease diagnosis by**	**Number of practitioners (%)**
Body external features and complaints	106 (75.7%)
Medical reports	6 (4.3%)
Nadi (pulse)	28 (20.00%)

% - Represent calculated percent value of traditional practitioner in each category.

It was observed that knowledge of the practice, other than from ancestors, was acquired from saints, relatives, friends or practitioners who treated them earlier for similar conditions. Further growth of traditional practices was through trial and error method or as an accident. Panda and Rout [43] reported finding of plant for bone-setting by traditional practitioner from Andhra Pradesh, as one such example of accidental discovery. Even the codified Ayurveda system adopted timely updates of knowledge from non-codified traditional practitioners [44]. Traditional practitioners indicated that learning of practice from forefathers begins normally at the age from 10 to 20 years. Learning starts with assisting the master in treatment, collection, cleaning and processing of medicinal plants, attending to preliminary complaints of patients and distributing medicines as directed by the master. This learning process continues for about 10–15 years before the novice can acquire usable knowledge of the practice. The knowledge transfer may be within the same family, same community or to an interested outsider. If new or younger generation is not interested in learning, the whole knowledge is lost with practitioner’s death.

### Diagnosis

Disease diagnosis is an important step leading to proper treatment and faster recovery of patients. Every medical system has its own methods for disease diagnosis. Table 2 shows the pattern of disease diagnosis followed by traditional practitioners of the district.

76% of traditional practitioners examine external body features and symptoms for diagnosis along with details provided by the patients. Different practitioners set their own unique parameters for assessment and identification of various diseases. For instance, in the diagnosis of jaundice, traditional practitioners check patients early in the morning on an empty stomach for change in eye colour (yellowish nature) and also by placing the juice of drum stick leaves on palm to see the change in its watery nature. In cases of snakebites, the practitioners check for changes in the tongue and tasting, whereas in case of herpes they diagnose by observing the nature of blisters/sores and their pattern. However, every practitioner asks for details regarding the symptomatic complaints and difficulties from patients. They clarify their doubts, if any, by cross questioning.

In cases like diabetes, blood pressure, HIV-AIDS, cancer, kidney problems and bone fractures, it was also observed that 4% of traditional practitioners use modern diagnostic tools and reports for their confirmation and then prescribe treatment. Traditional practitioners refer to the reports and statements of modern diagnostics for sugar level in blood and urine in case of diabetes, presence and type in HIV-AIDS and cancer, and size and location of the calculi in kidney stones. Diagnosis of bone fracture is based on x-ray reports, as documented by Upadhya et al. [12], in North-central region of Western Ghats of India.

The other category of practitioners (20%) use “Nadi Pariksha” for diagnosis, which can be correlated with pulse examination (‘Nadi’ = pulse and ‘Pariksha’ = examination). Though the term can be correlated with pulse examination in general, it may refer to nerves, veins and any kind of channel for passage of physiological and biological signals. It is an important technique of diagnostics in Ayurveda [32], and is followed also by allopathic practitioners [3]. Similar to Ayurveda, the traditional practitioners also define three humors viz. “Vata” (air), “Pitta” (fire) and “Kapha” (water). The practitioners evaluate for prognosis and diagnose the diseases by interpreting the imbalance in pulse pattern (“Nadi”), contributing to vitiated conditions of Vata, Pitta and Kapha [45]. This requires good knowledge of diseases, proper training and skill for interpretation. Generally “Nadi” is examined at the wrist immediately below the thumb, but examination of the same in different parts of the body like foot, both sides of neck, joints of hands and legs is also practiced by the traditional practitioners of this region like done in Ayurveda [46]. Efforts have been made to understand the system of “Nadi pariskha” in modern scientific terms, which showed positive correlation with disease diagnosis [47].

### Treatments

Traditional practitioners claim treatment for various diseases and illnesses, which were documented during the study. Among 140 traditional practitioners, 40 practitioners were specialized in the treatment of a single disease, 25 practitioners claimed to treat all kinds of diseases, while remaining claimed to treat two or more conditions. Depending on their specialization, the practitioners can be clustered as general practitioners who treat two or more kinds of diseases, bone setters, poison and bite healers, practitioners for mental health, spiritual practitioners and practitioners specialized for a single condition like jaundice, herpes, etc. Among the specialized practitioners for a specific or single condition, whole body poisoning locally known as “Kappa or Kuppatta”, was treated by 21% of practitioners. However, further scientific description of the condition, its etiology and investigation is needed for understanding its nature and importance. It was also noted that the traditional practitioners claimed effective treatment for common cold, cough and fever (36 practitioners); acute and chronic conditions like asthma (7), dysentery (25), herpes (5), jaundice (10), diabetes (20), kidney stone (8), HIV (7) and cancer (8). These claims gain attention as these conditions are reported to be prevalent in the region [48].

### Patient consultation and remuneration

**Figure 2:**
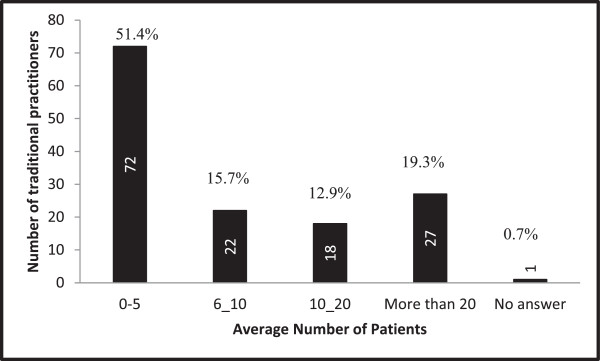
Average number of patients seen by traditional practitioners.

A larger number of patients from the region showed faith in traditional medicine and its efficacy (Figure 2), as reported earlier for other regions [49]. It was observed that 51% of traditional practitioners attend up to 5 patients per week, whereas 32% of practitioners treat more than 10 patients per week. In accidental cases like snake bite, dog bite, etc., the average number of patients per week was less than one and such practitioners treat a maximum of 5 to 10 such patients in a year. Many of these practitioners practice only on a fixed day in a week, which might be resulting in lesser number of patients. However, it was also observed that large number of patients, sometimes more than 100 (5%) visit the practitioner on a fixed day.

Traditional practitioners listen to all the complaints of patients, preferably in their own language. It was noted that about 30-40% of practitioners know other languages, such as Marathi, Hindi, Konkani along with Kannada, which is the local language. This helps practitioners to communicate on diets, do and don’ts, cautions and suggestions more effectively. Labhardt et al. [50] observed, traditional practitioners use a more patient centered communication style making patients comfortable and at ease. This patient friendly interaction not only soothes the patient’s psyche, but also makes the practitioner and the practice more popular amongst the public.

In this modern era, medical care is increasingly commercialized and is comparable to any business. In contrast, traditional medicine is still practiced more or less as a social service. Present study confirmed the same as 79% of traditional practitioners practice as a hobby or by interest with nominal or no monetary expectations from the public (Table 3). It was observed in the present study that, traditional practitioners (79%) have other main occupations for their livelihood, as reported earlier [35]. Therefore, normally they practice only on selected days, as per their convenience and beliefs. Nearly 70% of the practitioners in the study region practice on Wednesdays and Sundays. Practices on Tuesdays, full moon days and new moon days were also observed. It is observed that, only 15% of the practitioners practice it as their main occupation.

**Table 3:** Practicing kind and type of remuneration by traditional practitioners

**Remuneration**	**Number of practitioners practice as (%)**			**Total**
	**Hobby**	**Occupation**	**Other**	
**Money**	54 (38.6%)	21 (15.00%)	08 (5.7%)	83 (59.3%)
**Materials ** **(coconut, rice, etc.,)**	15 (10.7%)	Nil	1 (0.7%)	16 (11.4%)
**Free service**	41(29.3%)	Nil	Nil	41 (29.3%)
**Total**	110 (78.6%)	21 (15.00%)	09 (6.4%)	140 (100.00%)

(%) - Represent calculated percent value of traditional practitioner in each category.

29% of traditional practitioners conduct practice as a service to society. If someone forces them to take any kind of remunerations, they graciously advise to donate the same to a nearby temple or to some needy person. It was also noted that 11% of practitioners take remuneration in the form of rice, coconut, oil, etc., which is an Indian custom. However, a majority of traditional practitioners (59%) receive remuneration in the form of money. Most of the times, amount received is the actual cost of the medicine or with a small profit. Amongst these, 59% of the practitioners receive money as remuneration, 25% do practice traditional medicine for their livelihood.

### Mode of medicine preparations

Traditional practitioners use both plant and animal sources as their medicine. 98% of the practitioners use plant source as medicine, while none of the practitioners use only animal sources in their preparations. It is found to be an ancient tradition to use plants and animals as sources of medicine, not only in this region, but across various parts of the world [51-53].

Traditional practitioners collect medicinal plants in sufficient quantity, whenever they are available. 12% of the practitioners follow certain rituals before collection of the plants, which differ from practitioner to practitioner. The collection day varies from month (“Masa”), fortnight (“Pakshya”), day of the week (“Dina”), time (“Kaala”), star (“Nakshatra”) etc. It was also observed that, practitioners fix the time (“Muhurtham”) for plant collection and accordingly they follow the rituals. The rituals include taking bath in cold water early in the morning, worshiping god or spirit, wearing white or orange cloth, facing specific direction (generally North or East) during collection, keeping complete silence till collection and observing fasting during collection. Similar traditions have been observed earlier in Sikkim [38] and Maharashtra [3] states in India. Similar methods of medicinal plant collection are described in Ayurvedic texts in detail, which is substantially upheld by modern science that collection time, season and place has an effect on the chemical composition of the plant, which in turn affect its medicinal property [54]. This indicates the close relationship between Ayurveda and non-codified traditional medicine.

Among the practitioners, 70% store medicinal plants and dispense to patients as per the need. 17% of the practitioners collect fresh plants or plant parts for their usage, while remaining were not clear about storage. Various parts of the plants like leaves, seeds, fruits, root, stem, flowers, etc., are stored for future use for seasonal and/or rare plants. This kind of storage of drugs was reported earlier [12]. The storage methods need to be seen critically as storage conditions play a vital role in deciding safety and efficacy of the resultant medicine.

Traditional practitioners prepare various forms of medicine like decoction, paste, poultice, juice, etc. which vary from case to case. The same was suggested to patients for continuation during the course of treatment. Upadhya et al. [12,13], also observed similar type of medicine preparation in this region. Processing of medicinal plants with cow milk, ghee, cow urine, rain water collected on specific day, etc. was also observed. It was briefed by the practitioners that this treatment removes toxicity and/or enhances the medicinal property of plant material.

Animals and their products have constituted part of the traditional medicinal practice in various cultures since ancient times [53]. Traditional practitioners of this region commonly use cow milk, butter milk, ghee and cow urine in treating various disorders. 27% of practitioners use animal products as medicines (other than products from cow) along with plant medicines in the treatment. They also use milk of goat, buffalo or camel, hair, horn, bones or nails of domestic and wild animals like elephant, bear, bison, fox, deer and cats for different purposes, which was documented earlier by several workers [36,51-53,55].

### Magico-religious and spiritual practices

India is known worldwide for its spiritual culture. Traditional medicines have reported links with spiritual and magico-religious rituals in the country [56]. During the present study it was observed that, only two traditional practitioners practice religious rituals for treating patients. Both practitioners are in the age group of 71 and above and had practicing experience of more than 30 years. These practitioners are locally called “Baba” (saint) and patients visit them on particular days like new-moon and full-moon days. The practice includes chanting of prayers (“Mantras”), along with application of sacred ash (“Bhasma”), tying of holy threads to the patients, etc. Similar rituals have also been observed earlier in Tamil Nadu state of India for treating psychiatric patients [57]. Local people have faith in such practices. It was observed that large number of patients visits them on certain special occasions according to the Hindu calendar. Nonetheless, mixtures of magico-religious practices for the treatment of disease have been reported among various tribes throughout India [57-59]. On similar lines, the Yoruba medicinal practice of Africa consists of an inseparable union of magic, medic, and the mystic [60], which in turn can be compared to Nigerian [61], Spanish [62], and Iranian [63] magico-religious practices for treating ailments. Even though belief towards these practices is high in the local community, the credibility and science behind this needs to be addressed for their validation.

### Availability of plant resources and traditional conservational strategies

Usage of medicinal plants has increased over a period of time in the society. Therefore the demand for medicinal plants in the wild, which serves as the main source, is increasing. Degradation of biological diversity, fragmentation and various human interferences added to the fast depletion of medicinal plant resources, resulting in scarcity of medicinal plants.

From a conservation point of view it is important to note that about 90% of traditional practitioners collect medicinal plants from the wild, the practice of which is similar elsewhere [12-14,33,36]. Therefore, the present study reiterates the urgent need for protection and propagation of naturally available plant resources. 46% of traditional practitioners said some of the plant species are no longer available in the wild. They also opinioned that herbaceous plants which grow as weeds are in plenty, whereas shrubs and tree species are fast eroding and are difficult to get. Overall 66% of practitioners responded that availability of medicinal plants has declined over the years.

Further, preference ranking test was carried out, where practitioners ranked reasons for unavailability of medicinal plants according to their importance (Table 4). According to the practitioners, deforestation is the main cause for non-availability of medicinal plants followed by reduced rainfall, dam construction, forest fires and road widening. According to traditional practitioners opinion deforestation is primarily due to expansion of cultivation land, firewood collection and grazing. Similar causes were observed by Hegde et al. [64] for the decrease of medicinal plants in Belgaum district. Several conservational strategies were also recommended for medicinal plant management, which can be adopted by governing bodies of villages and traditional practitioners [64].

**Table 4:** Preference ranking test

**Reasons for**	Total score *(number of traditional practitioners)						**Overall ranking**
**Non-availability of medicinal plants**	**Ranking**					**Total**	
	**1**	**2**	**3**	**4**	**5**		
Deforestation	350	116	36	32	13	547	**1**^**st**^
	(70)	(29)	(12)	(16)	(13)	(140)	
Lesser rain fall	150	224	99	14	14	501	**2**^**nd**^
	(30)	(56)	(33)	(7)	(14)	(140)	
Dam constructions	100	96	177	28	23	424	**3**^**rd**^
	(20)	(24)	(59)	(14)	(23)	(140)	
Forest fire	75	64	69	152	10	370	**4**^**th**^
	(15)	(16)	(23)	(76)	(10)	(140)	
Road widening	25	60	39	54	80	258	**5**^**th**^
	(5)	(15)	(13)	(27)	(80)	(140)	
**Disinterest of younger generation in traditional medicinal practice**							
Higher education	215	252	45	30	4	546	**1**^**st**^
	(43)	(63)	(15)	(15)	(4)	(140)	
Migration from villages	250	136	153	4	3	546	**1**^**st**^
	(50)	(34)	(51)	(2)	(3)	(140)	
No attractive income	160	120	51	96	13	440	**2**^**nd**^
	(32)	(30)	(17)	(48)	(13)	(140)	
Difficulty in learning and practice	70	48	132	86	27	363	**3**^**rd**^
	(14)	(12)	(44)	(43)	(27)	(140)	
Non-availability of medicinal plants	5	4	39	64	93	205	**4**^**th**^
	(1)	(1)	(13)	(32)	(93)	(140)	

Ranking 1, 2, 3, 4, and 5 has the score of 5, 4, 3, 2, and 1 respectively; *Number of traditional practitioners gave the ranking for each category; Total score is sum of scores in each category; Overall ranking is with respect to descending order of total score.

Lack of availability of medicinal plants hinders the practice, which is also noticed elsewhere in India [33] and other parts of the world [55]. It is also true that the demand created by traditional medicine is one of the causes for over-exploitation of wild resources of medicinal plants [55], and this needs to be checked with awareness and afforestation programs. It is worth noting that practitioners of the region have good knowledge of medicinal plants [12,13] and have their own strategies for conservation. Traditional practitioners suggest their patients and friends grow the plants that are required. Practitioners also suggested cultivation of prioritized native plant species to meet their growing needs.

### Sharing of traditional knowledge

**Figure 3:**
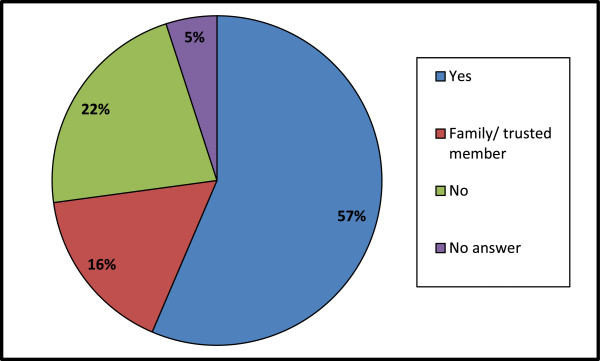
Traditional practitioner’s opinion about sharing of traditional knowledge.

Knowledge can survive and prosper only through dissemination and sharing. It is more so in case of traditional medicinal practice, because of no proper documentation. Existing knowledge needs to be transferred from the older generation to the next, which is possible only through sharing. It is generally believed that traditional practitioners are conservative and not ready to share their knowledge. Keeping this in mind the status of unwillingness towards knowledge sharing among practitioners was evaluated (Figure 3). It is surprising as much as inspiring to note that 57% of traditional practitioners were ready to share the knowledge with anyone, while 16% of practitioners were willing to share the knowledge only with trusted people like their family members. The remaining 22% of traditional practitioners were unwilling to share their knowledge, which can be a big hindrance in conserving valuable traditional knowledge.

Even though a large number of practitioners are ready to share their knowledge, there are hardly any takers. As the younger generation has little interest in learning this practice, it is resulting in fast erosion of age old traditions. Similar trend has been observed globally [65]. A preference ranking test was conducted to know the reason for the disinterest of the youth. According to traditional practitioners, it is mainly due to higher education and migration from villages to cities. The low income potential has made the younger generation to look to other opportunities with higher incomes. In addition, as mentioned earlier, the learning process itself takes more than ten years which makes it even less attractive. Medicinal plants are collected from the wild and their non-availability may be the other reason for the decreasing interest in the younger generation. These issues to be addressed at the grass-root level to revitalize traditional medicinal practices and to make it attractive to the younger generation.

From the above observations, and comparison with available earlier literature, it is evident that the present status of traditional medicine is more or less similar throughout India and various parts of the world. This practice is facing serious threats to its survival. Present regulatory policies are not fully capable of defining traditional medicine in terms of quality control with respect to medicine or practice. Hence, the global scientific community and policy makers need to look into the issues to understand traditional medicine as a whole in order to overcome the constraints in traditional medicinal practices and maximize its utilization.

### Constraints to traditional medicinal practice

India has well developed codified systems of medicine over the centuries. In India, regulation and systematic functioning of these codified traditional systems of medicine, starting with education at university level, registration of qualified practitioners, production, safety and efficacy issues of the drugs, are monitored by Department of Ayurveda, Yoga, Unani, Siddha and Homeopathy (AYUSH), Government of India [66]. However, in the presence of a strong codified traditional medicine, the non-codified system has lost its importance though there is high usage by the public [4]. It is also true that non-codified traditional medicine or folk medicine lack precise policy issues in India [67].

The problems start with the identification and registration of the practitioners. The traditional practitioners are not even recognized as healthcare providers by authorities in spite of their significant contribution to the system [4].

The traditional practitioners in the area opinioned that the issues of concern are fast depletion of this knowledge; inadequate legal frameworks for protecting their rights; lack of recognition and registration; lack of awareness among practitioners on regulatory issues and problems in procuring raw materials. Few of these issues were reported earlier from various parts of India [3,4,67]. Most of these issues need to be addressed at national levels. Meantime, flaws in the system like malpractices in the name of traditional systems, fake practitioners, nonexistence of strict quality control tools for drug materials and medicine, adulteration and substitution, and above all lack of scientific evidence for the safety and efficacy of these medicines are hindering further progress and promotion of traditional systems of medicine.

### Regulation and efforts for conservation of traditional medicinal practices

In India, even though the regulation of traditional system of medicine is still in its infancy, several initiatives have been taken to improve the system. They include Acts and Amendments like Biological Diversity Act 2002, Biological Diversity Rules 2004, Scheduled Tribes and other Traditional Forest Dwellers (Recognition of Forest Rights) Act 2006. These policies, instead of being concrete and elaborative, address only a few issues of traditional practitioners and their practice. Moreover, as these laws are nonspecific and covers various other aspects, there is a need to develop “sui*-*generis” laws for governing the rights of traditional practitioners [68].

The establishment of Traditional Knowledge Digital Library (TKDL) has facilitated the registry for traditional knowledge, which is a big step forward towards protection of the knowledge from bio-piracy. TKDL has thousands of Indian traditional formulations from traditional systems of medicine [69], which is available online for referencing and to deal with IPR issues. Still, the major portion of Indian non-codified traditional medicine remains undocumented and unprotected, paving way to bio-piracy. At the same time, intellectual property rights of non-codified traditional medicine practices are unprotected mainly because of their collective ownership and perpetuity development pattern from generation to generation which made the system “not so novel” commercially [68]. Kaushal [70] observed that strong patent protection has not been conducive to indigenous people and their traditional medicinal knowledge. She also added that the production and dissemination of legal clauses promoted by the Indian patent system is not an adequate legal tool for the protection of traditional medicinal knowledge. Therefore, firm and strong initiatives are required, both at the national and international levels, to protect the intellectual property rights of traditional practitioners.

Other important initiative for the recognition of traditional practitioners in India has been taken up by Indira Gandhi National Open University (IGNOU) through Centre for Traditional Knowledge Systems (CTKS). The prime objective of CTKS is to strengthen the traditional knowledge systems existing in different parts of India and empower the local communities across the country. A pilot project for “Certification of Prior Learning and Knowledge of the Gram Vaidyas” (Non-codified traditional village health practitioners) was launched in eight states across the country. Further process to establish procedures for validation and accreditation of the Gram Vaidyas is in progress in collaboration with Department of AYUSH, Ministry of Health and Family Welfare and National Rural Health Mission [34]. Several Non-Government Organizations such as Foundation for Revitalization on Local Health Traditions (FRLHT), Covenant Centre for Development (CCD), National Innovation Foundation (NIF), etc., are also working at regional level and joining hands with Government to ensure the well being of practitioners and also to preserve, validate and promote the traditional medicinal knowledge [71]. Institute of Ayurveda and Integrative Medicine (IAIM) has Centre for Local Health Traditions which supported creation of a network of over 250 Taluka-based traditional practitioners association across seven states of India to strengthen the non-codified practices.

These initiatives are only small efforts towards protecting non-codified traditional medicine looking for major initiatives by State and Central government. There is still a long way to go before the full benefits of this age-old knowledge can provide required healthcare, beyond the boundaries of various systems of medicine, to the community and help to achieve the global slogan of ‘Health for all’.

## Conclusion

Traditional medicinal practice in the region is at the crossroads of extinction, as a good number of practitioners are in the old age group. The practitioners use various techniques to diagnose the disease in the region including “Nadi Pariksha”, which needs further studies to establish the relations between non-codified traditional system and codified systems of medicine like Ayurveda. The traditional practitioners claim effective treatment for different types of illnesses using plant and animal sources as medicine. Fast depletion of medicinal plants is the main concern in traditional practice, which needs to be addressed through conservation and sustainable utilization. Although the traditional practitioners have showed interest in transferring the knowledge to the next generation, the younger generation is not interested in acquiring it because of their higher educational status and poor income potential of the practice. Hence conservation strategies for both the knowledge and resources are the need of the hour. As the existing scientific parameters, legal instruments and social mindsets do not meet the actual needs of traditional practices, tailor made systems need to be established by the main stakeholders to prove the science of traditional medicine, to safeguard the interest of practitioners and to conserve the knowledge.

## Competing interests

Authors declare no competing interest.

## Authors’ contribution

VU contributed to conduct the study, acquisition, analysis of data and scientific literature search. VU, HVH and SB contributed to interpretation of data, and developed and drafted the report. SB and SDK contributed in study design and approved final draft of the report. All authors reviewed and approved the manuscript.

## Supplementary Material

Additional file 1 Interview Schedule for Traditional practitioner. Click here for file
