# Supplementary material for: Non-codified traditional medicine practices from Belgaum Region in Southern India: present scenario
Source: J Ethnobiol Ethnomed. 2014 Jun 16;10:49. doi: 10.1186/1746-4269-10-49 (PMC4084578; doi:10.1186/1746-4269-10-49)
Supplement: Additional file 1 — Interview Schedule for Traditional practitioner. [file 1746-4269-10-49-S1.docx]

**Interview Schedule for Traditional practitioner**

1. Name of the Practitioner with address
2. Age (in years), Sex, Religion
3. Experience (in years)
4. Place of practicing
5. How you learnt the practice?
6. Practicing as (Occupation, hobby, etc.)
7. Is this your full time profession?
8. Mention the diseases you generally treat.
9. Which disease you are specialized in treating?
10. What is the Mode of diagnosis?
11. Average number of patients seen per week?
12. Did you use medicinal plants? Yes/No
    1. If yes, Please provide the details
13. Do you get the plants in sufficient quantity for your practice? Yes/No
    1. If No please provide reasons
14. Do you prescribe any animal product for treatment / prevention of diseases? Yes/No
    1. If yes please provide the details
15. Are you ready to share / teach the knowledge of herbal medicine to others?
16. What kind of remuneration you are getting from the patients?
17. Preference raking test:
    1. Non availability of Medicinal plants

| **Question** | **Non availability of Medicinal plants** | | | | |
| --- | --- | --- | --- | --- | --- |
| **Reasons** | Lesser rain fall | Deforestation | Road widening | Forest fire | Dam constructions |
| **Ranks form ( 1-5)** |  |  |  |  |  |

- 1. Non interest of younger generation in traditional medicinal practice

| **Question** | **Non interest of younger generation in traditional medicinal practice** | | | | |
| --- | --- | --- | --- | --- | --- |
| **Reasons** | Higher education | Non availability of medicinal plants | No attractive income | Migration from villages | Difficulty in learning and practice |
| **Ranks form ( 1-5)** |  |  |  |  |  |

**Note:** *Each question is followed by detailed open end discussion*
